# The use of multispectral imaging to distinguish reactive urothelium from neoplastic urothelium

**DOI:** 10.4103/2153-3539.71064

**Published:** 2010-10-11

**Authors:** Christopher Michael Gilbert, Anil Parwani

**Affiliations:** Department of Pathology, University of Pittsburgh Medical Center, Pittsburgh, PA, USA

**Keywords:** Multispectral imaging, urothelial neoplasia

## Abstract

**Context:**

The interpretation of urothelial atypia in a setting of chronic inflammation and reactive changes can prove difficult with small biopsies. Limited recuts lessen the efficacy of ancillary studies such as CK20, P53 and CD44 in these instances.

**Objective:**

To evaluate a triple-immunostain with the assistance of multispectral microscopy.

**Design:**

Fifty-three bladder biopsies with previous diagnosis of benign/reactive, dysplastic, carcinoma in situ or carcinoma were prepared using a tripleimmunostain cocktail consisting of CK20, P53 and CD44. Three control stains were used for the purpose of creating a spectral library for the Nuance CRI Flex microscopy system. All specimens were interpreted by light microscopy, processed with the Nuance 2.71 software, and CK20 and P53 were scored blinded to the case diagnoses. CD44 was not scored as it proved difficult to interpret in many cases.

**Results:**

The results demonstrated that it was possible to separate CK20, P53 and the counterstain that were co-localized in the biopsies. Separation of the stains demonstrated a correlation of p53 and CK20 dual expression in biopsies diagnosed as carcinoma. Low or undetectable levels of expression were seen in biopsies later diagnosed as reactive or benign.

**Conclusion:**

The combination of multispectral microscopy and multiple immunostain cocktails form a powerful and useful tool for the interpretation of small biopsies with faint or difficult to interpret staining and for cases with limited material such as small-bladder biopsies.

## BACKGROUND

The advantages of immunostains were apparent in 1941 when Albert Coons first described their use.[1] In the years since, immunostains have been applied to varied techniques such as flow cytometry, Western blotting, enzyme-linked immunosorbent assays and electron microscopy in diverse fields including histopathology, forensics, laboratory medicine and cell and molecular biology. Immunohistochemistry is probably the most evident technique where they are used to identify and characterize cells, in many cases with high sensitivity and specificity. Despite their numerous advantages they are limited in at least one crucial way. The chromogen substrates used to produce color in most commercially available immunostains are rather limited in their spectral specificity and diversity. It is intuitive, therefore, that the staining of tissue with more than two antibodies becomes increasingly difficult for the human eye to interpret and attempt to quantify.

Multispectral imaging is a technique that captures image information across a broader range of the electromagnetic spectrum than the human eye and traditional RGB cameras. It permits the analysis of tissue with greater spectral densities and resolution of varied overlapping chromogens. There are applications in nearly every subspecialty area of surgical pathology for which using multiple antibodies on the same section would be of great value. This is doubly important in the evaluation of small biopsies where the preservation of scant tissue is sometimes essential. In these instances cutting additional tissue levels carries the risk that the area of interest will no longer be present on the tissue to be stained. The interpretation of small, inflamed or reactive needle-core urothelial biopsies with CK20, P53 and CD44 is one such setting.

### Aim

Our aim is to evaluate a triple-immunostain consisting of CK20, P53 and CD44 with the assistance of multispectral microscopy in order to, in a single slide, evaluate their simultaneous expression and location in urothelium, and, at the same time, diminish the risk of losing the area of interest in further recut sections.

## METHODS

After institutional board approval, 53 formalin-fixed and paraffin-embedded bladder biopsies with previous diagnoses of benign/reactive, dysplastic, carcinoma in situ or carcinoma (approximately 39 cases spanning 1998-2006) were retrieved. Four micrometer sections from each were obtained and incubated with a triple-immunostain cocktail composed of CK20, P53 and CD44 antibodies. The CK20 (DAKO, Carpinteria, CA - Ks20.8), P53 (DAKO - CM1) and mouse monoclonal CD44 (Cell Signaling Cat#3570 - 156-3C11) antibodies were added in 1:100, 1:500 and 1:600 concentrations, respectively. Van Gogh yellow diluent Cat# PD902L was added as a diluent and Harris hematoxylin as the counterstain. Biocare Medical chromogens Cardassian DAB Brown Cat#DBC959H, Vulcan Fast Red Cat#FR803S and Bajoran Purple Cat#BJP811L were utilized for the CK20, P53 and CD44 antibodies, respectively.

**Figure 1:**
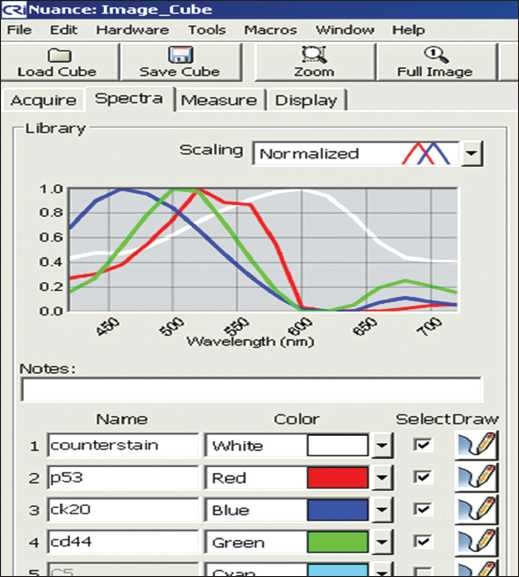
Spectral range: white-counterstain, blue-CK20, green-CD44 and red-p53

From the control stains a spectral library was created for the Nuance CRI Flex microscopy system (spectral range of 420-720 nm). This spectral library is seen in Figure 1. It was built by sequentially adding each control’s specific wavelength and subtracting the counterstain. The bright-field mode was used for analysis. All specimens were captured and analyzed with the custom-built spectral template and analyzed using the provided Nuance software version 2.71 with the use of programmable macros that automated the majority of the work once properly setup. The macros auto-calibrated, captured and separated the image “cubes.” The cases were scored blinded to the diagnoses using light microscopy. CK20 was given a score of “1+” if it involved the upper 1/3 of urothelium, “2+” for 2/3 of the urothelium and “3+” for full thickness staining of the urothelium. P53 was scored as “0” or negative, “1+” or weak, “2+” or moderate and “3+” or “heavy.” CD44 was not scored as it proved difficult to analyze and interpret in many cases due to poor staining.

## RESULTS

**Figure 2:**
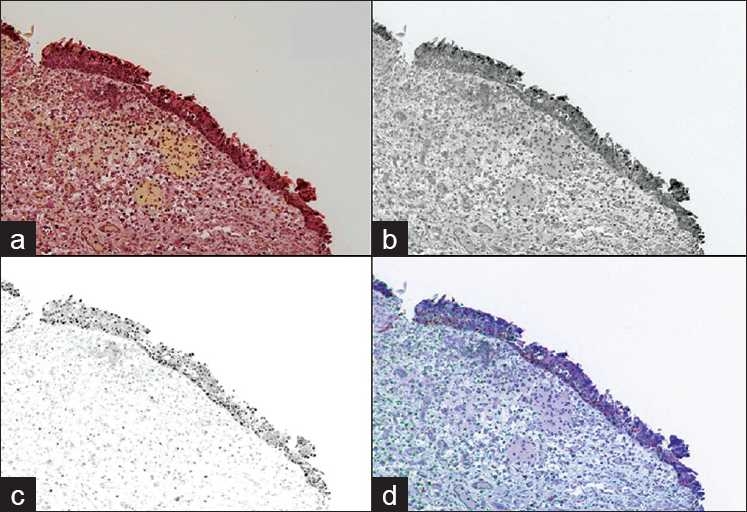
(a) shows a traditional photographic image (RGB) of the slide. Following “unmixing,” (b) demonstrates CK20 staining limited to the umbrella cells/upper 1/3 of the urothelium and (c) shows the P53 staining. (d) shows a composite of the unmixed stains re-assigned with different colors for easier interpretation. This case was graded as 2+ for P53 and 1+ for CK20. The diagnosis given was reactive urothelium

The results, shown in Table 1, demonstrated that it was possible to separate the multiple stains co-localized in the biopsies, including the counterstains. Separation of the stains demonstrated a correlation of p53 and CK20 dual expression in biopsies diagnosed as carcinoma. Low or undetectable levels of expression were seen in biopsies later diagnosed as reactive or benign. For benign or reactive cases, CK20 was restricted to the upper 1/3 in 17/23 biopsies (74%) and p53 was weak or negative in 11/23 biopsies (48%). No benign or reactive biopsies stained heavily (3+) for p53. Figure 2 provides an example of a case diagnosed as “reactive.”

**Table 1:** The urothelial biopsies are stratified by diagnosis and show increasing p53 and CK20 expression with malignancy

Diagnosis	P53 (2,3+)	%	CK20 (2,3+)	%
Benign or reactive	12/23	52	6/23	26
Dysplasia	2/6	33	2/6	33
CIS or carcinoma	19/26	73	19/26	73

**Figure 3:**
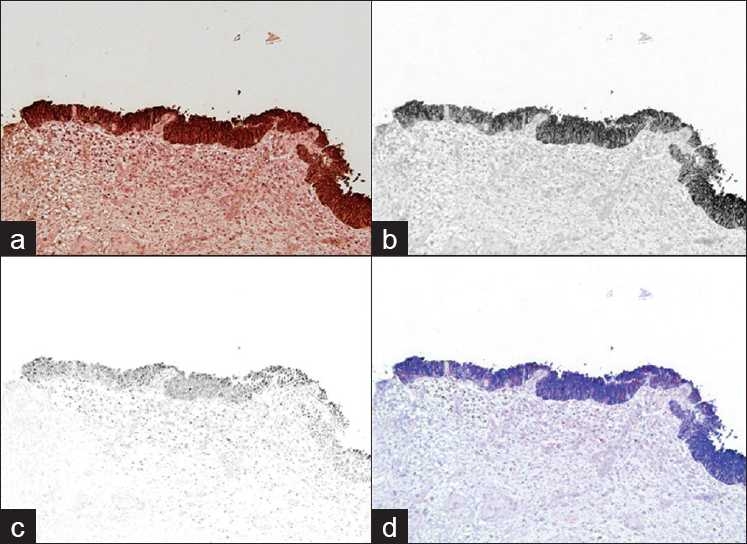
(a) shows a traditional photographic image (RGB) of the slide. Following “unmixing,” (b) demonstrates CK20 staining the full thickness of the urothelium and (c) shows the P53 staining. (d) shows a composite of the unmixed stains re-assigned with different colors for easier interpretation. This case was graded as 2+ for P53 and 3+ for CK20. The diagnosis given was urothelial carcinoma in situ

For cases of carcinoma in situ or invasive disease CK20 was expressed in the upper 2/3 or full thickness of the urothelium in 19/26 (73%) of cases while p53 was moderate or heavy in 19/26 cases (73%). Figure 3 shows an example of a case diagnosed “urothelial carcinoma in situ.” Cases diagnosed as “dysplastic” appeared to resemble benign reactive biopsies with only 2/6 (33%) expressing moderate or heavy p53 and CK20 restricted to the upper 1/3 in 66% (4/6) of the biopsies.

## DISCUSSION

As the number of immunostains increase on a slide, particularly when by design they overlap, co-localization and accurate interpretation becomes more difficult. From a laboratory perspective, these “cocktails” can be difficult to design given a limited number of wavelength options, variable staining intensity and inevitable background staining. These limitations in combination make interpretation of such slides difficult and time consuming with the naked eye. Multispectral image analysis is one purported solution for this problem that has been recently studied in fine needle aspiration of the thyroid[2] and examined in a review of quantitative imaging techniques.[3]

The staining results presented here are consistent with past studies at the University of Pittsburgh Medical Center performed with an equivalent cocktail.[4] They support patterns of p53 and CK20 expression that vary by diagnosis as reported previously in the literature.[5,6] Subjectively, the addition of multispectral imaging permitted much easier interpretation aided by enabling the manipulation of the staining patterns. The use of programmable macros greatly improved the efficiency of capturing that information accurately and quickly. Built into the Nuance software are tools, not presented here, that permit objective quantification of staining density and co-expression, worthy of future investigation. Future improvements not limited to enhanced spectral density, accommodating larger number of immunostains, enabled by combining fluorescence mode in combination with quantum-dots[7] may provide a level of sophistication to immunohistochemistry not yet seen and ripe for future investigation.

As this study was a pilot, there are a number of important limitations worthy of discussion. CD44 expression was poor in the cocktail batch; an unanticipated result given the success with it in previous studies. In selected cases, bright-field analysis was able to compensate to a sufficient extent to reliably discriminate the three stains despite the spectral similarity between the CD44 and P53 chromogens. This was the exception rather than the norm, and therefore, felt to represent anecdotal evidence of Nuance’s ability to manipulate 3 stains under bright-field examination, but requires further scrutiny. Even with appropriate staining, the spectral signatures of the chromogens used with the CD44 and P53 antibodies were overly broad with considerable overlap. Quantum dots have been shown to provide much narrower spectral signatures and would make an interesting comparison. Further investigation is also needed to compare the results from Nuance’s multispectral camera to a “spectral unmixing” RGB method, as described by Ruifrok *et al*.[8]

Lastly, the value of compressing multiple slides into a single tissue section was of limited benefit. With rare exception, extra blank slides cut with the first facing of the block would have sufficed. However, one situation where this value may be realized is an unexpected finding on a cytologically obtained cell block, a setting that invites further investigation.

In conclusion, the combination of multispectral microscopy and multiple immunostain cocktails form a promising tool for the interpretation of cases with limited material such as urothelial needle core biopsies. This is true moreover for applications that favor immunohistochemical stains without the need for fluorescent markers or the expense of confocal microscopy. The fields of cytopathology, hematopathology and surgical pathology will serve as prominent subjects for further experimentation in the future.
